# Can outcomes of dyadic interactions be consistent across contexts among wild zebrafish?

**DOI:** 10.1098/rsos.150282

**Published:** 2015-11-04

**Authors:** Tamal Roy, Anuradha Bhat

**Affiliations:** Department of Biological Sciences, Indian Institute of Science Education and Research Kolkata, Mohanpur, West Bengal 741 246, India

**Keywords:** winner–loser, aggression, courtship, zebrafish, food monopolization

## Abstract

Winner–loser relations among group-living individuals are often measured by the levels of aggressive interactions between them. These interactions are typically driven by competition for resources such as food and mates. It has been observed in recent studies on zebrafish that dominant males generally have higher total reproductive success than their less aggressive subordinate counterparts. This study aimed to test whether males who monopolized a food resource (winners) also displayed higher levels of aggression than the males who were unsuccessful (losers). Further, the study also tested whether the same ‘winner’ males were also able to monopolize interactions with females during courtship. The results from these experiments showed that while males monopolizing food resources (winners) demonstrated higher levels of agonistic interactions than the losers, the average number of courtship interactions initiated by either of the males (i.e. winners/losers) with a female was not significantly different. A significant relationship was obtained between the number of aggressive interactions and feeding latencies of males in the context of food monopolization. This indicated that there could be a linkage between boldness defined by feeding latency in a novel environment and agonistic responses. The probable role of nature of resources, resource availability and distribution in determining the outcomes of dyadic contests is discussed.

## 1. Introduction

Group-living organisms display a variety of social interactions that are sometimes cooperative (such as schooling, kin recognition and social learning) or agonistic (such as territoriality and competition for mates) in nature [1]. Agonistic behaviour, often resulting in direct aggressive interactions among individuals in social environments, has several important implications to their survival and fitness [2]. Many species of group-living fishes have been shown to employ agonistic interactions to establish dominant–subordinate relationships [3–5]. Such interactions are often employed during competition for common resources (food, mates or shelter) within the group. The nature of the resources and their availability would therefore be important in determining agonistic response and establishment of dominant–subordinate relationships among group-living individuals. However, once established, consistency of these dominant–subordinate relationships across different contexts of competition (i.e. kind of resource) in a group has not been explored so far. This study deals with the investigation of winner–loser relationships among wild zebrafish across contexts in a dyadic combat. In fishes, aggression has been shown to depend on social status with dominant individuals displaying higher levels of aggression indicated by greater bites and chases than subordinate individuals [6,7]. Aggressive behaviour facilitates the competitive defence of key resources (food, territory and mates) and allows formation of clear dominant–subordinate relationships, with respect to access to such resources [8,9]. Owing to their higher aggressive displays, dominant individuals have a greater access to food than their subordinate counterparts. The consequence of such relationships becomes more pronounced in the context of mating when the dominant individuals, by virtue of higher aggressiveness can monopolize territories as well as potential mates while the subordinates are deprived [10]. Social status as being dominant/subordinate has also been known to affect sperm production resulting in dominant males with better sperm quality than subordinates (cichlid fish, *Astatotilapia* (*Haplochromis*) *burtoni*) [11]. Therefore, dominant individuals have higher chances of passing on their genes to the next generation, influencing mating or reproductive success [12].

Zebrafish is a group-living species with definitive social assemblages (shoals) and this gregariousness predisposes it to exhibit behaviours that are developed from interactions, aggressiveness or agonistic response being one of them. Aggressive interactions (comprising chasing and biting) are typically directed towards conspecifics for establishing and maintaining dominance hierarchies [5,13–15]. Factors such as density of conspecifics, social-experience, relative size of individuals and environmental conditions can also affect aggression in zebrafish [16–19]. Aggression is also important during foraging and feeding—more dominant individuals often succeed in monopolizing food over less dominant conspecifics [20–22].

Consistency in winner–loser relationships have been previously demonstrated in species such as Mozambique tilapia [23] and zebrafish [2]. Indeed, consistent behavioural differences in terms of agonistic interactions can exist among individuals and further, these can be observed across contexts, as has been recently shown through repeated mirror trials and dyadic interactions of male green swordtails, *Xiphophorus helleri* [24]. This study investigates whether individuals win across contexts of competition for two of the most important resources, food and mates, that is, whether individuals that win in a given context are also successful in another behavioural context. Specifically, the study tests whether an individual male that is the winner with respect to food monopolization in the presence of a conspecific male (i.e. in a dyadic set-up) also (i) displays greater aggression against the conspecific male, and (ii) displays a greater number of interactions with a female in the presence of the same male during courtship and mating. We measured the male–male interactions during food monopolization in the form of chases and bites, and also recorded the identity of the male that was first able to eat the food. We quantified the number of male–female interactions in the form of nudges and pushes (characterizing courtship) during mate monopolization. We hypothesized that males who won the food monopolization contest would be more aggressive (i.e. inflict greater number of chases and bites) than males who lost. Also, males who are winners in the food monopolization contest would display a greater number of interactions with a female as against the males who lost. We referred to the outcomes of a single contest for each kind of resource for all pairs of males and denoted the individuals of the pairs as ‘winners’ or ‘losers’ on the basis of these outcomes. This is in agreement with the popular definitions of results of such contests between pairs of individuals [25].

## 2. Material and methods

Wild population of zebrafish (*Danio rerio*) were collected from a stagnant water body (a ditch adjacent to paddy fields) in Nadia district of West Bengal (India) for the behavioural study. Wild-caught individuals were brought to the laboratory and housed in bare holding tanks (45.7×25.4×25.4 cm) consisting of standard corner filters, for acclimatization. Holding room temperature was maintained at 27°C and lighting conditions at a 14 L : 10 D (hours) cycle to mimic natural conditions essential for courtship and spawning. The fish were fed standard pellet food, freeze-dried bloodworms and *Artemia* (brine shrimp) alternately, once a day (morning). To ensure full-grown adults for the experiments, the wild-caught fish were reared in the laboratory for at least six months before the start of experiments.

Prior to the start of behavioural tests, 80 males and 40 females were sorted from the population and kept separately in six different stock aquaria (30.5×20.3×20.3 cm), each holding 20 individuals, until the females became gravid. During this period (about two weeks), fish (males and females) were fed freeze-dried bloodworms once a day (morning), to ensure that they get habituated to this food resource. Out of these, 60 males (to form 30 pairs) and 30 females were selected for the experiment. Each of the two males of a pair (selected from different stock aquaria) used for a test was matched for size. The identity of each male of the size-matched pair was noted based on body coloration, stripe pattern and physical state (fat/thin) in order to differentiate between them during subsequent experiments and video analysis. This method of identification based on distinguishing body features has been reliably used in earlier studies [12]. Individual fish to be used for trials were kept in clearly labelled separate, 1000 ml cylindrical plastic containers (filled with 520 ml water) for social (visual and chemical) isolation, for a period of minimum 7 days [26]. Following social isolation, each pair of males was subjected to tests for food and mate monopolization. The two kinds of tests were interspersed with a social isolation period of minimum 7 days. The order of the mate and food monopolization tests was randomized for the pairs.

### 2.1 Competition for monopolizing food

**Figure 1.**
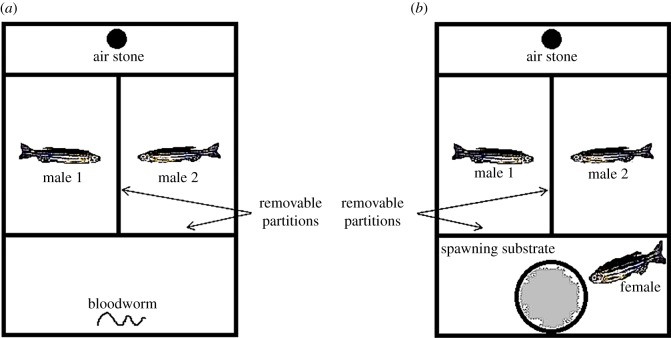
(*a*) Schematic outline of the design of experimental tank for the food monopolization experiment. (*b*) Schematic outline of the design of experimental tank for the mate monopolizationexperiment.

A bare glass tank (30.5×20.3×20.3 cm) with opaque removable partitions to constitute four chambers was used for our experiments (figure 1*a*). The narrow compartment (20×6 cm) at the rear end consisted of an air stone and the opaque partition separating this chamber from the middle one was perforated to allow for aeration throughout. The broad (20×13.5 cm) middle compartment was further divided into two chambers (10×13.5 cm) which were perpendicular to the front-end compartment (20×10 cm; figure 1*a*). The sides of the tank were lined with brown paper to prevent fish in the tank to see those in the other.

Tests were conducted on 30 pairs of males, in batches of 10, on three consecutive days. The experiments were performed during the morning hours when fishes were normally fed. The fishes were starved on the day of the actual experiment to equalize their hunger levels. The experiment tank was filled with well-aerated water to a depth of 13 cm, and provided an air stone for constant aeration. Water temperature was maintained at 27°C throughout the experiment. A pair of (size-matched) males was first introduced to the experiment tank—each male put in one of the two adjacent chambers and allowed 10 min for acclimatization to the environment. At the commencement of the trial, the opaque partitions separating the two males from each other and the perpendicular chamber (empty) was gently raised. A piece of freeze-dried bloodworm was then slowly dropped near the centre of the front compartment of the tank. The worm was dropped when the fish were approximately at the same distance from the front-edge of the tank and to ensure equal visibility to both the fish. Previous studies have used a 5 or 10 min observational window for testing establishment of dominant–subordinate relationship [9,27,28]. The present experiment was carried out for 10 min and was video recorded on a high definition (HD) digital camera (Canon PowerShotA3300 IS) to avoid possible disturbance to the fish owing to an observer. The use of a HD digital camera facilitated successful distinction between males constituting a pair during video analysis by picking up the physical differences between the same. After the experiment, the males were identified (based on the physical characteristics of the individuals previously noted such as pattern or shade of the stripes, slight difference in body shape, etc.) and put back into their respective labelled containers, for social isolation for at least a week. The experimental tank was thoroughly cleaned and refilled before the next pair of males was tested.

The HD videos were analysed at reduced speed (0.5×) in order to distinguish between individual males of a pair. From the video-recordings, the identity of the first of each pair to grab and eat the bloodworm was noted. This male was assigned the status of a winner and the other, the loser. The latency to feed was also recorded for the fish. The fish which were unsuccessful in eating the food were assigned an upper limit value of 600 s (10 min) as the feeding latency. We also recorded the number of aggressive responses by each male of a pair. In zebrafish, the acts of aggression comprised biting, nipping and brief bouts of fast-swimming towards an opponent [29]. The number of chases and bites by each male towards its counterpart was thus counted for agonistic encounters.

### 2.2 Competition for monopolizing mate during courtship

A similar experimental tank set-up (as described above) was used for conducting the mate monopolization (figure 1*b*). The same pairs of 30 males as in the previous food monopolization experiment were used in the competition of monopolizing a potential mate. Thirty females (one female each for a pair of males) were selected from the stock (main holding tank) for the tests. Zebrafish are typically known to spawn and mate in the early hours of the day (i.e. daybreak) [30]. Therefore, all the trials were conducted during the early morning window at 08.00 when the lights went on (i.e. during artificial ‘daybreak’). On the evening before the experiment, the experiment tank was filled with well-aerated water (to a depth of 13 cm) and fitted with an air stone for constant aeration as previously described. Water temperature was maintained at 27°C. As in the other experiment, a pair of size-matched males was transferred to the adjacent chambers (with opaque partitions in between them and also with the front chamber) in the experiment tank. A spawning site consisting of a Petri plate with gravel was placed in the front chamber and a female was transferred into this chamber (figure 1*b*). The following morning, at ‘daybreak’, the opaque partitions separating the males from each other and from the female were raised and the fishes were video-recorded for 10 min using a HD digital camera (Canon PowerShotA3300 IS). After the experiment, the males were identified based on the specific physical features (e.g. shade of stripes, slight difference in body shape, etc.) noted previously, separated and transferred back to their labelled holding containers. The females were put back into the stock tanks. We removed the Petri plate (the spawning site) and checked for eggs in order to ensure that successful spawning had happened. The trials in which no eggs were found on the spawning plates were discarded and another test trial for the same pairs of males was repeated (following the same protocol that included isolation followed by the trial) using a new female.

The videos were analysed at reduced speed (0.5×) in order to distinguish between individual males of a pair. Previous studies have characterized the courtship behaviour in zebrafish males and females in detail [31,32]. The male typically chases a female by swimming side by side, nudging it with its snout, encircling around it leading to spawning [32]. From the video recordings of the trials, such male–female interactions in the form of nudges/pushes that led to spawning were counted individually for each male constituting a pair and these interactions were subsequently used as the parameter for quantifying monopolization of a female by a male.

### 2.3 Statistical analyses

All data analyses were conducted using StatistiXL (v. 1.8) software and R v. 3.1.1 package. Based on the results of the food monopolization experiments, the males of each pair were categorized as winner and loser. Of a pair, the winner male was the one which was successful in grabbing hold of the food, whereas the loser the one which lost in the competition. Subsequently, the number of male–female interactions during courtship (mate-monopolization experiment), and the number of aggressive interactions during food monopolization, were assorted for the ‘winner’ and loser’ categories. The data on number of male–female interactions by the winner and loser males in the mate-monopolization experiment were first tested for normality using the chi-square goodness-of-fit test. The number of male–female interactions by the winners and the losers was compared using the Student’s paired *t*-test. Comparison of aggressive interactions initiated by winners versus interactions initiated by losers was also conducted using Student’s paired *t*-test.

In order to determine the relationship between food monopolization and aggressive interactions, feeding latencies were compared against the number of aggressive interactions for all the males (*n*=60) using Pearson’s correlation. To control for potential confounding effects of paired males in the experiment, we constructed a mixed model (using the lmer function in lme4 (v. 0.999375-31) package [33] in R (v. 3.1.1) with observation identification (ID) (or each test trial between a pair) as random effect, aggressive interactions as fixed effects and latency to feed as the response variable. We employed the Kenward–Roger approximation for measuring the degrees of freedom [34]. The relationship between feeding latencies and number of male–female interactions for the males was also explored using Pearson’s correlation. Again, a mixed model was constructed with observation ID as random effect, male–female interactions as fixed effect and latency to feed as the response variable.

## 3. Results

The data on the male–female interactions in the paired trials were found to be normally distributed (chi-squared test $\chi_{6}^{2}=9.14$, *p*=0.16 for ‘winners’ interactions and $\chi_{5}^{2}=10.7$, *p*=0.06 for ‘loser’ interactions). Thus, paired parametric tests were performed for the subsequent analyses.

**Figure 2.**
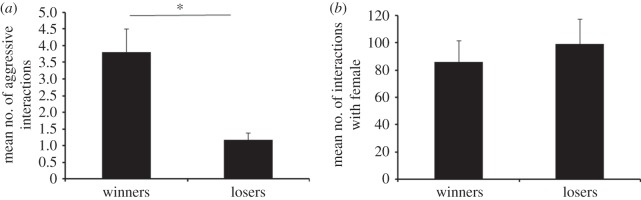
(*a*) Comparison of the mean number of aggressive interactions initiated by either of the males (winners versus losers). Error bars indicate standard error of means. Significant difference (*p*<0.01) is indicated by an asterisk. (*b*) Comparison of the mean number of interactions initiated by either of the males (winners versus losers) towards the female during courtship. Error bars indicate standard error of means.

There was a significant difference in the mean number of aggressive interactions displayed by the winners and losers (paired *t*-test: *t*_28_=3.63, *n*=30, *p*<0.001; figure 2*a*). There was no significant difference between the mean number of male–female interactions initiated by the two categories of males, the winners and losers (paired *t*-test: *t*_28_=−1.23, *n*=30, *p*=0.11; figure 2*b*).

**Figure 3.**
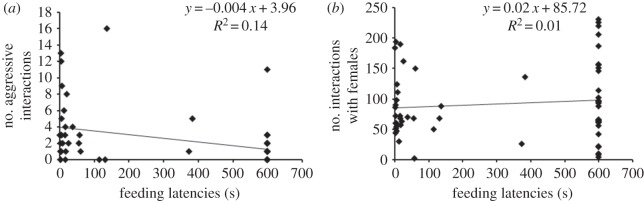
(*a*) Relationship of feeding latencies with number of aggressive interactions for males (*n*=60) during food monopolization (*r*=−0.375, *p*=0.003). (*b*) Relationship of feeding latencies with number of male–female interactions for males (*n*=60; *r*=0.101, *p*=0.44).

Feeding latencies were negatively correlated with number of aggressive interactions for the males used in the experiment (*r*=−0.375, *n*=60, *p*=0.003; figure 3*a*). The linear-mixed model (with each dyad pair as random intercept) showed a significant negative relationship between feeding latency and aggressive interactions (estimate for aggressive interactions=−30.80; s.e.=±10.0; *t*_138.4_=−3.08; *p*<0.00). There was no significant correlation between feeding latencies and number of male–female interactions (*r*=0.101, *n*=60, *p*=0.444; figure 3*b*). Linear-mixed model (with each dyad pair as random intercept) for a relationship between feeding latency and male–female interactions also showed no significant relationship (estimate for male–female interactions=0.48; s.e.=±0.63; *t*_136.3_=0.77; *p*=0.44).

## 4. Discussion

The results from the study indicated that the winner of the competition for food monopolization was also more aggressive than the loser in the competition for food. In addition, there was no significant difference between the male–female interactions of winners or losers during courtship. In other words, the males who won the competition for food did not necessarily interact or court more with the female and vice versa. A negative relationship was obtained between the number of aggressive interactions and the feeding latencies of males. Additionally, there was an absence of a significant relationship between the number of male–female interactions and feeding latencies of all males taken together. Our results are based on preliminary observations on dyadic interactions that test whether there is consistency in relationships across pairs. However, additional experiments that test repeated responses within each pair would help in establishing the existence of consistent winner–loser relationships within pairs.

The proximate cause for mismatch observed in the status of males of a dyadic pair during food and mate monopolization contests could be attributed to the differential hormonal circuits controlling behaviour of males in these two occasions. Previous studies in zebrafish have documented relationships between boldness and aggressive behaviours [16,26]. In our study, we found that the individuals who were successful in the food monopolization contest also displayed higher levels of aggression than the ones who were defeated. Latency to feed in a novel environment is a test commonly used to measure boldness. Here, the latency of males to feed, in general, was negatively correlated with the number of aggressive interactions imparted by each male in a pair towards its counterpart. Our results seemed to be in intuitive agreement with the study by Dahlbom *et al*. [26] that the outcome of a dyadic fight can be predicted from tests for boldness, with bolder individuals being more likely to become dominant. Therefore, it could be argued that the males who were successful in monopolizing food could have been bolder than the males who were defeated in the competition for the same. On the contrary, we did not find any significant relationship between the feeding latencies of males and the number of male–female interactions. This was in support of the mismatch found in the winner–loser outcomes of the dyadic contest which showed that the individuals who fed first (and therefore had lowest feeding latencies) did not necessarily court a female more.

This study aimed to test whether aggressive males, while benefiting in terms of their access to females were also ‘overall’ successful in terms of other factors such as monopolization with respect to food resources. It is speculated that this could be due to the difference in nature of the resource for which the competition occurs, i.e. food resource or mate resource. Resource availability and its distribution can affect social animals that forage in groups and dominance status can predict access to restricted food sources [35]. Aggressive responses among individuals have been seen to increase with clumped or limited resource availability [36]. The responses could depend on whether the resources are distributed uniformly or unevenly in space [37]. Where resources are limiting, individuals that are consistently competent would be expected to have an advantage over incompetent individuals. For example, spatial clumping is known to increase food monopolization and its defence among convict cichlids [38]. A continuum concept of social organization (differing in resource access and distribution) suggests that changes in resource distribution can produce a wide range of responses and predictable variation among individuals [36,39,40]. In this study, where the food resource is limited and unevenly distributed, competition for this resource resulted in clearer aggressive displays. However, in the case of competition for a mate, the interaction of both the males with the female is less skewed towards any particular male. In natural environments, the quality and distribution of the food resource would be expected to be more uneven than the distribution of mates (in this case, females).

Higher levels of aggression have been demonstrated to be vital for reproductive success—male zebrafish defend territories and restrain the entry of subordinates thereby preventing their access over the spawning sites as well as females [13,14,29,31,41]. Dominant males are successful in siring more offspring than the subordinates, thereby accounting for higher reproductive success [12,42]. Additionally, the preference of the female is an important driving factor for successful courtship and spawning. The females might exert a choice for courtship and hence might accept advances from a certain male while refuse to respond to the other. A combination of these factors could account for the variability in winner/loser status for the males in zebrafish with respect to monopolization of two kinds of resources.

This study indicated that the difference among competing males was not apparent at least at the courtship stage as measured by the interactions of each male with the female (the number of nudges and pushes with the female) during courtship. It could be possible that not all visible nudges/pushes resulted in successful fertilization, because the female might have had preferentially spawned more (i.e. in greater numbers) when a particular male was closer, leading to more successful fertilization of eggs by the same. Ecological determinants such as population density along with operational sex ratio can also play a substantial role in the context of male territorial aggression and tend to have a definitive impact on females in terms of egg production [41,43].

In conclusion, we have demonstrated that the results of dyadic combats involving males of zebrafish for resources such as food and mates could yield different outcomes. The basis of these outcomes could be differential endocrine mechanisms governing the behaviours, the linkage between correlated behaviours or the basic nature of resource. Further investigations on female preferences and choice across populations with varying sex ratios along with assays of endocrine activity associated with these responses are warranted.

## Supplementary Material

S1. Number of aggressive interactions initiated by Winners and Losers of the food monopolization tests

## Supplementary Material

S2. Number of interactions (with the female) initiated by the Winner and Loser (male) individuals of the food monopolization tests

## Supplementary Material

S3. Feeding latencies of each male with corresponding aggressive interactions (with the other male) and interactions with females

## Data accessibility

Data on the aggressive interactions (i.e. between male pairs) and number of interactions with females (i.e. of each winner and loser male) are provided in the electronic supplementary material, S1 and S2, respectively. Data on the feeding latencies of each male along with corresponding aggressive interactions (with the other male) and interactions with females are provided in the electronic supplementary material, S3.
